# The Politics of Fear: Médecins Sans Frontières and the West African Ebola Epidemic

**DOI:** 10.3201/eid2311.171206

**Published:** 2017-11

**Authors:** Keith Hamilton

**Affiliations:** University of Pennsylvania, Philadelphia, Pennsylvania, USA

**Keywords:** Ebola, Ebola virus, viruses, epidemic, crisis response, politics of fear, Médecins Sans Frontières, West Africa

The Politics of Fear: Médecins Sans Frontières and the West African Ebola Epidemic is an engaging collection of essays that critically evaluates the response of Médecins Sans Frontières (MSF) to the Ebola epidemic in West Africa during 2014 and 2015. It is an appropriate mixture of reflections, vignettes, case studies, and critiques regarding the role of MSF in the epidemic. It does not offer detailed background on the organization itself or on the Ebola epidemic. Thus, the target audience includes readers who understand the role of MSF, as well as the basic principles of Ebola virus disease (EVD). The book describes complexities of management of such a large outbreak and provides context for the difficult decisions made by MSF leadership, as well as clinicians on the ground. Many books and articles have been written about the epidemiology of EVD, but this book is unique in its focus on the politics and ethics of caring for EVD patients in a complex sociopolitical environment.

The content is well balanced, and chapter authors include MSF physicians, MSF nurses, health officers, administrators, social anthropologists, and bioethicists. MSF as an organization is presented as neither savior nor villain in the crisis; it is described objectively as a player whose actions were motivated by the epidemic itself and its decisions as difficult choices at a time where there were no right choices. The essays eloquently describe the motivations behind such decisions as quarantine of patients, participation in clinical trials, allocation of resources, evacuation of medical personnel, and use of experimental treatments. Each account provides a unique perspective of different aspects of the epidemic and response. In these descriptions, the authors highlight the complicated dynamics in navigating limited resources, overwhelming disease, and citizen distrust. Permeating these essays is the humanity and courage of the doctors, nurses, and staff caring for patients.

This book achieves its goal of describing the multifaceted considerations that must be taken into account when designing policy and response to an epidemic and highlighting the vulnerabilities of healthcare systems that must be addressed to achieve more effective response. It also raises the argument that global humanitarianism, although motivated by altruism, must adapt to existing economies, cultures, and politics if there is any hope for a successful outcome. Although the description of the MSF response is powerful, the essay structure of the multiauthored book does not necessarily provide a coherent approach or strategy to avoid and more effectively treat future crises. However, the book does an outstanding job of outlining the problems that must be addressed.

This book is not a detailed description of the science or epidemiology of EVD but provides in-depth explanation and assessment of the actions of MSF in response to the Ebola epidemic. It is an appropriate resource for those readers who wish to better understand the intricacies of a global epidemic humanitarian response in the context of the disparate sociopolitical environments in which they exist. The reader will appreciate the courage of MSF healthcare providers in the response to the Ebola epidemic, as well as the problems that must be addressed to ensure the dedication and sacrifice of these providers translate to favorable outcomes. 

